# Antibacterial resorbable barrier membranes with therapeutic activity for guided tissue regeneration

**DOI:** 10.1590/1807-3107bor-2025.vol39.119

**Published:** 2025-11-07

**Authors:** Larissa Faria SILVEIRA, Fabricio Mezzomo COLLARES, Vicente Castelo Branco LEITUNE, Rosane Michele Duarte SOARES, Gabriela de Souza BALBINOT

**Affiliations:** (a) Universidade Federal do Rio Grande do Sul – UFRGS, School of Dentistry, Dental Materials Laboratory, Porto Alegre, RS, Brazil.; (b) Universidade Federal do Rio Grande do Sul – UFRGS, Institute of Chemistry, Polymeric Biomaterials Laboratory, Porto Alegre, RS, Brazil.

**Keywords:** Guided Tissue Regeneration, Anti-Bacterial Agents, Polymers

## Abstract

The objective of this study was to develop a bioabsorbable membrane composed of poly(butylene adipate-co-terephthalate) (PBAT) with alkyl trimethyl ammonium bromide (ATAB) to provide antimicrobial properties. Membranes were manufactured via the solvent casting technique using chloroform solutions containing PBAT and varying concentrations of ATAB (1, 2.5, and 5% wt), with ATAB-free membranes as a control. The characterization of the membranes included assessments of contact angle, surface free energy, and degradation in distilled water over periods of one week, one month, and three months. Mechanical properties were evaluated via tensile strength, and changes in water pH were monitored from 24 hours to three months post-immersion. Cytotoxicity was assessed using gingival fibroblasts and pre-osteoblasts via the SRB assay. Antimicrobial activity was tested against Staphylococcus aureus. ATAB inclusion reduced water contact angle and increased surface free energy compared to controls (p < 0.001). The addition of 5% wt ATAB decreased the tensile strength of PBAT membranes. At one month, a reduced mass was observed for the 2.5% wt ATAB membrane. The specimens’ mass was reduced for all groups after three months of immersion in water in comparison to the initial measurement, while a reduction in thickness was found in all time points, without the influence of ATAB. ATAB incorporation reduced cell viability. Antimicrobial efficacy, resulting in a > 3 log_10_ bacterial reduction, was observed for *S. aureus* at the 5% wt concentration. The addition of 2.5% wt ATAB to PBAT membranes may be a suitable strategy to generate barrier membranes with an antibacterial effect while maintaining acceptable mechanical and surface properties.

## Introduction

Guided tissue regeneration (GTR) has been explored to recover or limit vertical bone loss after tooth extraction.^1^ Even though this technique aims to protect the alveolus during the initial period of alveolar repair, it is estimated that 16.8% of GTR procedures using barrier membranes fail due to post-surgical infection and acute abscess, which occur along with soft tissue dehiscence and membrane exposure.^2^ The presence of post-surgical infection after GTR with non-resorbable polytetrafluoroethylene membranes decreases the amount of bone that can be regenerated from 90-100% to 42-60% of the initial bone volume.^3^ One strategy to overcome problems involving bacterial infection is the development of membranes with local release of antimicrobial agents.^4-6^

Various polymers used in barrier membranes have been associated with antimicrobials.^4,5,7-10^ These polymers can be classified as resorbable or non-resorbable, synthetic, or natural. Among the synthetic polymers, aliphatic polyesters poly(lactic acid) (PLA) and polycaprolactone (PCL)^9,10^ are used as carriers for antimicrobials due to their controlled biodegradation, yet they are too stiff for this application. Aromatic polyesters have excellent mechanical properties but are resistant to degradation. Aliphatic-aromatic co-polyester, such as poly(butylene adipate co-terephthalate) (PBAT) combines the biodegradation properties of aliphatic polyesters with the mechanical properties of aromatic polyesters.^11^ This polymer has been studied for the fabrication of barrier membranes, as its structure combines flexibility and resorption capacity with stability and mechanical resistance, allowing it to be combined with antimicrobial agents.^12^

Quaternary ammonium compounds (QACs) are cationic surfactants that react with the phospholipid components of the bacterial plasma membrane, causing membrane damage and triggering events that lead to the lysis of microorganisms, including enveloped viruses, gram-positive bacteria, and gram-negative bacteria.^13,14^ Alkyl trimethyl ammonium bromide (ATAB) is a long-chain QAC with broad bactericidal and fungicidal action.^15^ ATAB addition to dental materials resulted in the inhibition of bacterial growth without altering the physical properties and biocompatibility of the materials.^16-19^ Moreover, its antimicrobial mechanism is known to act on several bacterial strains without eliciting resistance to its action. The combination of resorbable synthetic PBAT with ATAB for functionalized barrier membranes may be a strategy to produce tailored materials for guided bone regeneration (GBR). Thus, this study aims to develop a bioabsorbable membrane using PBAT with different concentrations of ATAB to determine the required concentration for the production of antibacterial membranes.

## Methods

### Barrier membrane production

The membranes were produced by solvent casting^12^. Poly(butylene adipate-co-terephthalate) (PBAT - Ecoflex^®^ F Blend C1200; BASF Corporation, Florham Park, USA) pallets were mixed in chloroform at 2 g:15 mL for 24h. The ATAB (Sigma Aldrich, St. Louis, USA) was added to the polymeric solution at 1, 2.5, and 5 wt%. PBAT membranes without ATAB addition were used as a control group. Casting took place on glass petri dishes, and the solvent was evaporated for 30 min in a 37°C incubator to obtain films that were used for the assays as prepared. Both ATAB and PBAT were imaged with scanning electron microscopy (SEM, TM300- Hitachi, Tokyo, Japan) at 15kV and 200x magnification and 10kV and 500x magnification, respectively.

### Contact angle and surface free energy

The sessile drop method was used to measure the contact angle with water (n = 5) and α-bromonaphthalene (n = 5) was used for polar and dispersive measurements.^12^ The membranes (10 mm diameter and 0.2 mm thickness) were positioned in glass slides and a high-resolution camera was used to monitor the behavior of the water drop on the material surface for 20 s, while the measurements of the contact angle were performed after 10 s by an image software (OneAttension, Biolin Scientific, Stockholm, Sweden). At 10 seconds, the image was analyzed to determine the contact angles between the tangent of the liquid and the solid on the left and right sides of the image. The average of these two angles was used as the measurement value. The surface free energy calculations were performed according to the OWRK/Fowkes theory, in which the contact angle is used to assess the interactions between the surface of a material and different liquids. With a known interfacial tension, it is possible to estimate the solid surface tension based on independent polar and dispersive components, and results are calculated and reported in mN/m.

### Mechanical behavior

A tensile test was conducted according to ASTM D6338 type IV plastics (n = 6).^20^ Hour-glass specimens were prepared with 115 mm length, 13 mm width, and 200 µm thickness. The constriction length was standardized at 50 mm. Before testing, the specimens were kept immersed in distilled water for 24 h so that the test was conducted with wet samples. A mechanical testing machine (Shimadzu EZ-SX, Shimadzu Corp., Kyoto, Japan) was used in tensile mode at a speed of 1 mm/min. Results were recorded in Newton and the tensile strength was calculated based on the cross-sectional area of each specimen.

### *In-vitro* degradation

Degradation was assessed via changes in membrane mass and thickness after immersion in water for up to three months.^13,21,22^ For mass measurements, the samples (6 mm diameter and 200 µm thickness) were weighted individually in an analytical scale, and a digital caliper was used to assess the membrane’s thickness. Changes in the pH of the distilled water in which the membranes were immersed were measured using a digital pH meter (DM 22, São Paulo, Brazil). The water in contact with the membranes was replaced every 7 days.

### Cytotoxicity

Cell culture was performed with the preosteoblastic MC3T3-E1 cell line (Banco de Células do Rio de Janeiro, RJ, Brazil) and with primary gingival fibroblastic cells.^12,23^ Cell proliferation was assessed by the sulphorodhamine B (SRB) assay with a direct-indirect method. Material extracts were produced with membrane specimens (6 mm diameter × 200 µm thickness; n = 5) immersed in a culture medium for 24h. Cells were cultivated at 5 × 10^3^ density in a 96-well plate, and treatments were performed for 72h. Cells were fixed and stained with 0.4% SRB solution. The SRB dye was quantified at 560 nm in a microplate spectrophotometer (Multiskan GO, Thermo Fisher Scientific, Waltham, USA). The absorbance in wells cultivated for the same amount of time without membrane immersion was used to normalize the results in wells with the immersion of the samples.

### Antibacterial activity

The antibacterial ability of membranes was tested against *Staphylococcus aureus* (ATCC 25923).^24,25^ The specimens (4 mm diameter and 200 µm thickness; n = 3) were immersed in 10% bacterial brain-heart infusion (BHI) inoculum for 24 h at 37°C. For biofilm quantification, three specimens were vortexed for 1 min to remove adhered microorganisms before dilutions were made up to 10^6^in 96-well plates. Dilutions were plated in BHI agar Petri dishes for 24h for colony formation. For planktonic analysis, the inoculum was collected from wells after contact with materials, and dilutions were made up to 10^6^. Bacteria were platted as described for the biofilm analysis for 24h for colony growth. In this case, a negative control without material immersion was used to assess the bacterial suspension. All analyses were conducted in aerobic conditions. The number of colony-forming units (CFU) was visually counted by optical microscopy and transformed to log_10_ CFU/mL.

### Statistical analysis

Normality was assessed by the Shapiro-Wilk test. One-way ANOVA was used to assess differences in surface properties, cytotoxicity, and antimicrobial data, with Tukey as the post-hoc for multiple comparisons. Two-way ANOVA and Tukey test were used to analyze the data on in vitro degradation over time. The analyses were performed at a significance level of 5%.

## Results

**Figure 1:**
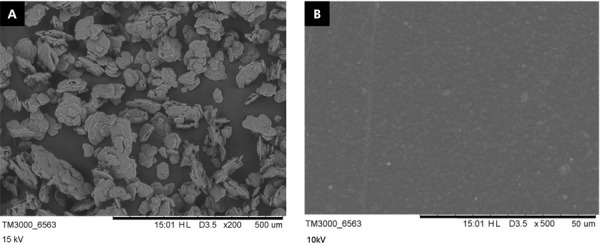
SEM images from (A) ATAB structure and (B) PBAT membranes.

ATAB morphology presented plate-like structures in SEM, while a flat surface was obtained by PBAT (Figure 1). The water contact angle was lower in the groups where ATAB was added (Table; p < 0.001), and no impact of ATAB was observed when α-bromonaphthalene was used (p = 0.601). This behavior led to differences in the surface free energy of developed membranes. ATAB promoted an increase in surface free energy when compared to the pure PBAT in the control group.

**Table:** Mean and standard deviation values of contact angle with water and α-bromonaphtalene and surface free energy (SFE) for the different concentrations of ATAB on the developed membranes.

Groups	Contact angle		Surface free energy
	Distilled water	α-bromonaphtalene	
PBAT	71.81 (± 5.52) A	16.88 (± 8.76) A	48.25 (± 2.62) B
ATAB 1%	9.77 (± 4.15) B	19.77(± 5.30) A	75.14 (± 4.96) A
ATAB 2.5%	17.59 (± 10.82) B	21.95(± 10.76) A	73.34 (± 4.99) A
ATAB 5%	10.74 (± 4.34) B	20.04 (± 5.23) A	76.12 (± 1.01) A

Different letters in the columns indicate statistically significant differences between the groups.

**Figure 2:**
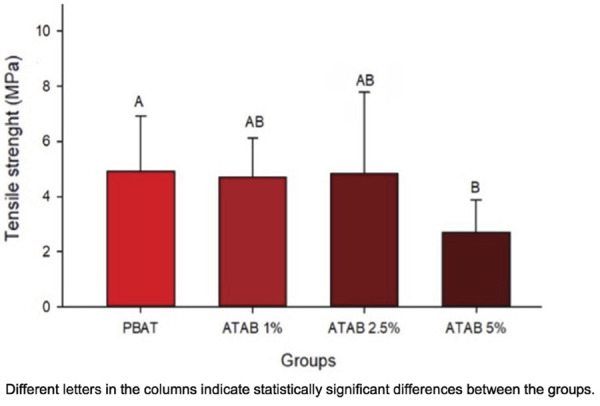
Mechanical behavior of developed membranes. Mean and standard deviation values for tensile strength of developed membranes.

**Figure 3:**
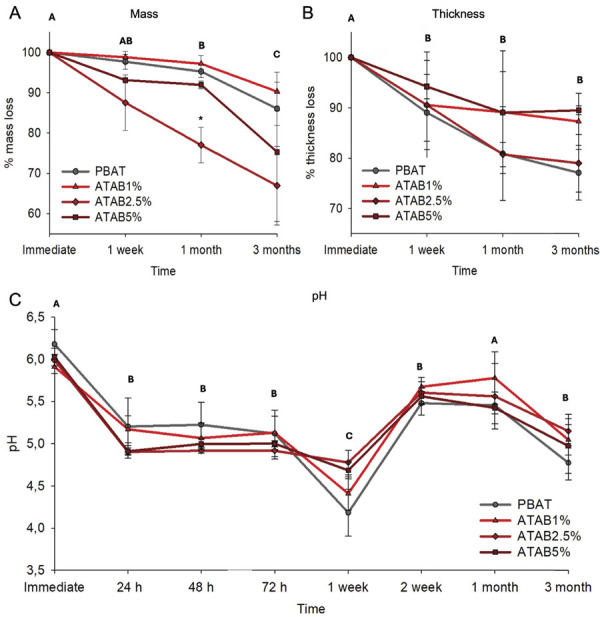
In vitro degradation of membranes. Percent reduction in mass (A) and thickness (B) after 1 week, 1 month, and 3 months in the different membranes added with ATAB and the control. Changes in pH are shown for up to 3 months in the water used for degradation assay (C).

The mechanical behavior of the developed membranes is shown in Figure 2, where a reduction in tensile strength was observed after a 5 wt% addition to the PBAT structure. No difference from the control was detected at up to 2.5 wt% of ATAB. In vitro degradation results are shown in Figure 3. The specimens’ mass was reduced in all groups after three months of immersion in water in comparison to the initial measurement (Figure 3A). The three-month mass value was also reduced when compared to one-week and one-month values (p < 0.05). No differences were observed between groups for thickness in the in vitro degradation analysis. Both time and materials affected the water pH (p < 0.001), which was reduced from the initial analysis to 1 week of immersion. The pH of water from PBAT membranes (control group) was significantly lower than that tin the ATAB-containing groups at one week and 2 months.

**Figure 4:**
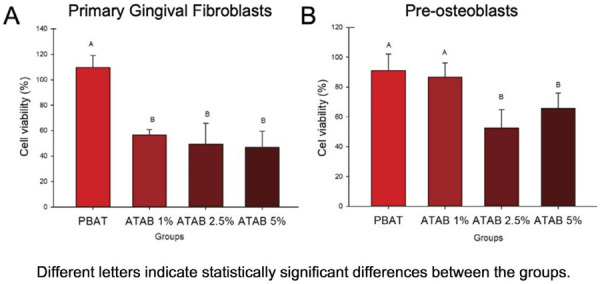
Cytotoxicity analysis in GBR-related cell lines. Mean and standard deviation values for SRB analysis of primary gingival fibroblasts (A) and pre-osteoblastic (B) cell viability in indirect-direct cytotoxicity analysis.

The viability of fibroblastic cells ranged between 46.85% and 56.56% in ATAB-containing membranes, while the control group presented 109.53% viability (Figure 4A). This reduction was similar for all concentrations of ATAB. In the pre-osteoblastic cell analysis, the 1% ATAB group had no significant difference from the PBAT group (p = < 0.001), with values higher than 86% of cell viability (Figure 4B). Higher concentrations (> 2.5%) caused a reduction in cell viability when compared to the control group.

**Figure 5:**
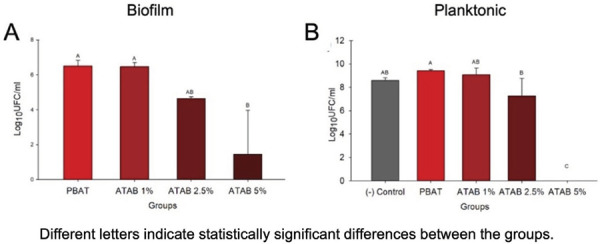
Antibacterial activity for biofilm and planktonic bacteria. Mean and standard deviation in Log10CFU/mL after contact of the membrane with *S. aureus* biofilm (A) and planktonic bacteria (B).

Figure 5 shows the results of the antibacterial analysis. Antibacterial activity was observed for Staphylococcus Aureus in biofilm with membranes loaded with 5% wt ATAB and in planktonic bacteria from up to 2.5% wt ATAB. The 5% wt concentration promoted a > 3 log_10_ reduction in bacterial colony counts and no colony was detected in planktonic analysis at 5 wt%.

## Discussion

The primary objective of an antimicrobial membrane is to help the regeneration processes by controlling the proliferation of microorganisms that may impair the healing of hard and soft tissues. The ATAB added to the PBAT membranes aims to provide antibacterial activity to inert membranes without affecting their physicochemical and biological properties. The surface of PBAT was modified by the addition of ATAB by enhancing its wetting and surface free energy, while reducing its mechanical strength. A degradation of the membranes was observed for all groups after one week, which was not modified by ATAB addition. This may be related to the reduction in cell viability promoted by these membranes and the observed antibacterial activity against *Staphylococcus aureus*, a prevalent bacterial strain for soft tissue infection in the oral cavity.

The addition of up to 1% wt ATAB leads to an amphiphilic behavior to the membrane’s surface.^26^ This is observed in the results from the contact angle with distilled water (Table). A reduction from 71.81° (± 5.52) in the pure PBAT membrane to 17.59° (± 10.82) in the 2.5% ATAB group was found, which evidences the affinity of ATAB by non-polar molecules, making the wetting more pronounced in these materials. The affinity for water led to the increase in surface free energy from 48.25 (± 2.62) in pure PBAT membrane to 73.34 (± 4.99) in the 2.5% ATAB group (Table 1). These changes may be assigned to the structure of ATAB and to possible changes in membrane surface due to the morphology of ATAB (Figure 1). A hydrophobic character is usually observed in synthetic polyesters that are used in the manufacturing of bone regeneration composites, and this could limit the interaction between materials and the surrounding tissues during repair. This interaction could be beneficial to the wetting of fluids, blood cells, and cells involved in wound repair ^27^. At the same time, the higher surface free energy can modify the bacterial adhesion on the membrane’s surface. Some bacterial strains in the oral cavity adhere more readily to materials with a higher surface free energy^28^and this could potentially enhance biofilm formation on membranes that are exposed to the oral environment, which is commonly related to failures in GBR treatments. While this could be a cause of concern, the antibacterial effect against *S. aureus* qA still observed IN the developed membranes, especially at higher ATAB concentrations (Figure 5A and 5B).

The addition of up to 2.5% wt ATAB did not affect the structure and the mechanical behavior of PBAT (Figure 2), while the 5% wt ATAB addition lowered the tensile strength when compared to the PBAT membrane (p < 0.05). This reduction is explained by the fact that ATAB does not copolymerize with PBAT.^29^ While a 54% reduction was detected when compared to PBAT alone, the values found for ATAB-containing membranes were comparable to commercially available membranes ^30^. Collagen is the most used material for resorbable membranes, and a reduced strength is found for these natural polymers, with tensile strength ranging between 0.16 and 2.24 MPa when membranes were tested in wet conditions,^31^ similar to this study. It is known that the repair of bone defects is favored by materials with higher mechanical strength, which can better maintain the space needed in GBR.^32^ While there is a large variability in the materials used for GBR membranes and the mechanical behavior of collagens are a drawback for its application in these procedures,^32^ this finding is not detrimental to the clinical application of the developed membranes.

The degradation of membranes over time may affect their ability to maintain the bone defect closed following the principles of GBR. A maximum 34% mass degradation was observed at three months when 2.5% wt ATAB was used (Figure 3A), with a difference between 2.5% wt and the other analyzed groups at one month. After three months, the addition of ATAB did not result in higher degradation of the membranes (p < 0.05) and > 60% of the membrane weight was maintained. The limited loss in membrane structure may be assigned to the PBAT structure and the assay characteristics. The degradation of synthetic polyesters is controlled by their crystallinity and chain structure.^13,33^ The stability in aromatic domains and the organization of aliphatic structures make these materials less prone to degradation than naturally-derived polymeric membranes.^21^ In collagen-based membranes, the reduction in mass can reach 90% at 50 days of water immersion, which is related to the high hydrolysis of its structure. While the time for in vivo degradation cannot be inferred by our results, there was little impact of ATAB in this process.

The degradation of PBAT may explain the changes in water pH after the immersion of membranes (Figure 3C). A pH reduction was observed in all compounds with a greater reduction for the control group at 2 months of immersion (Figure 3C). ATAB-containing membranes have a less pronounced acidic effect on the surrounding environment than PBAT membranes in this case. PBAT is derived from 1,4-butanediol, adipic acid, and terephthalic acid, and the pH reduction may be attributed to the release of acidic monomers during degradation or interaction with water ^34,35^ . The release of degradation products has not yet been shown for PBAT in medical devices, but a low concentration of phthalates has been found in non-medical applications, allowing the application of PBAT as a biodegradable polymer.^36^ This is consistent with the changes in pH observed in this study. Moreover, ATAB release could limit this acidic environment during the initial degradation of these compounds.

The release of ATAB may be the reason a cytotoxic effect was found in the developed membranes (Figure 4). This is related to the release of antibacterial compounds by the ATAB addition. The ATAB structure, especially in its free form, may present cell-damaging behavior, with rapid release and an increase of dose.^18^ The antibacterial mechanism that involves loss of membrane integrity may affect eukaryotic cells and lead to a reduction in their viability. ATAB may affect cell behavior in bone and soft tissues during the healing process in GBR procedures and limit the healing near the implanted membrane; thus, these results must be considered in the clinical application of these materials.

The threshold for in vitro analysis of cell cytotoxicity is 70%, as recommended by ISO 109973^37^ and even though this study did not conduct cytotoxicity experiments using the ISO parameters, the cell viability values were below 70%, which raises concerns about the application of ATAB-loaded PBAT membranes. Another possible cause of cell cytotoxicity could be the changes in pH (Figure 3). However, these do not appear to be related to a reduction in cell viability, as all groups presented similar values at an early stage, and no cytotoxicity was detected in the control group where PBAT was used alone. This confirms the biocompatibility of PBAT, as shown previously.^29^ Antibacterial compounds, such as QUACs, commonly present cytotoxic effects.^38^ Previous reports failed to find low cell viability results for ATAB when inserted into dental materials,^12^ but the use of its free form and the absence of interaction between ATAB and PBAT could make this effect more pronounced. However, the study used a 2D direct-indirect assay, and it is known that this analysis may overestimate the cytotoxicity results^39^ with no association to in vivo results. This cytotoxic effect may be studied further in pre-clinical analysis with low ATAB concentrations to ensure minimum effect on surrounding cells. To avoid the undesired effects of cytotoxic antibacterial compounds, ATAB is used in controllable systems for antibacterial compound delivery. Otherwise, the interaction between membrane components may be improved to ensure that antimicrobial membranes do not affect the ability of any involved tissues to recover during bone repair in GBR procedures.

An antibacterial effect was observed in membranes with ATAB in both biofilm and planktonic analysis (Figure 5). *S. aureus* was tested in this initial screening as it is a common bacterium in bone infections, responsible for most cases of osteomyelitis due to its ability to colonize the bone and produce numerous virulence factors.^40^ *S. aureus* is one of the first strains to colonize these areas, being responsible for biofilm formation. Thus, reducing its viability could limit the proliferation of other strains.^40^ This antibacterial effect can be attributed to the cationic agent in the ATAB molecule, which interacts with the negatively charged polar head of phospholipids in the bacterial plasmatic membrane, leading to the loss of structural organization and membrane integrity.^41^This causes progressive leakage of intracellular material, reducing bacterial viability and colony-forming ability. Thus, planktonic bacteria are affected and biofilm formation is reduced. Bacteria in the media are affected by the cationic agent, while the loss of viability due to membrane contact with ATAB prevents bacterial adhesion and the progression to biofilm organization. The 5% wt ATAB in the membrane reduced planktonic *S. aureus* by > 3 log10UFC/mL, indicating that the release of ATAB into the medium was sufficient to produce a significant antibacterial effect. Besides the planktonic effect, the formation of biofilm plays a significant role in sustaining the infection, leading to delayed wound healing by triggering the recruitment of inflammatory cells.^42^ These factors disrupt the essential balance between osteoblasts and osteoclasts and limit the amount of bone that can be formed.^1^ As mentioned before, biofilm formation may be influenced by the surface free energy of the membranes, which enhances bacterial adhesion in the oral environment.^28^ This study did not investigate bacteria adhesion to the membranes. Thus, future studies could explore this further, along with investigating the antibacterial effect against multispecies biofilms, to better understand the antibacterial effects in complex conditions. Even though this was observed in Table 1, reduced the biofilm formation was found in 2.5 and 5 wt% ATAB-containing membranes, showing that the compound itself can help control of bacterial adhesion and biofilm growth in these membranes.

## Conclusions

PBAT/ATAb membranes were successfully produced. The addition of up to 5 wt% ATAB led to the creation of barrier membranes with antibacterial effects while maintaining acceptable mechanical and surface properties.

## Data Availability

The datasets generated during and/or analyzed during the current study are available from the corresponding author on reasonable request.
